# Supplementary material for: Silver Nanostars-Coated Surfaces with Potent Biocidal Properties
Source: Int J Environ Res Public Health. 2020 Oct 28;17(21):7891. doi: 10.3390/ijerph17217891 (PMC7662325; doi:10.3390/ijerph17217891)
Supplement: Supplementary file 1 [file ijerph-17-07891-s001.pdf]

International Journal of
Environmental Research and Public Health

MDPI

Supplementary Materials

Silver Nanostars-Coated Surfaces With Potent Biocidal Properties

Lucinda J. Bessa [1,¥,*], Miguel Peixoto de Almeida [1,¥], Peter Eaton [1], Eulália Pereira [1] and Paula Gameiro [1]

[1] LAQV/REQUIMTE, Departamento de Química e Bioquímica, Faculdade de Ciências, Universidade do Porto, 4169-007 Porto, Portugal

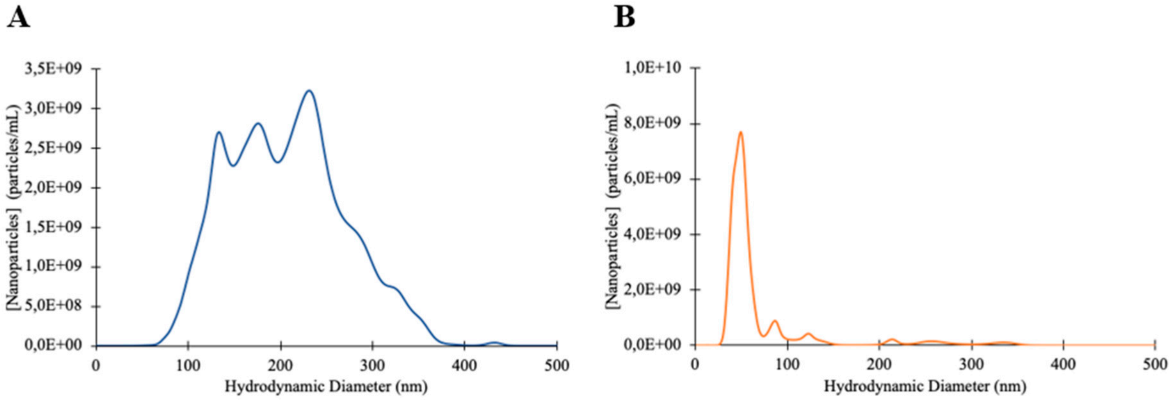

Figure S1. Hydrodynamic diameter distributions for AgNSs (A) and AgNPs (B), obtained by nanoparticle tracking analysis.

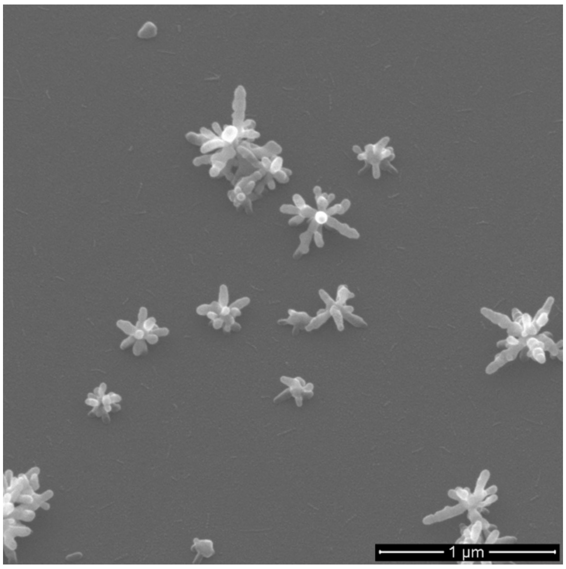

Figure S2. Scanning electron microscopy micrographs of AgNSs.

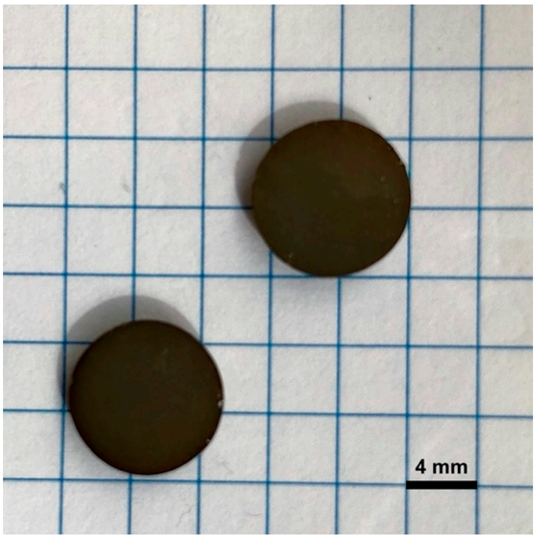

Figure S3. Photograph of the AgNSs-coated surfaces.

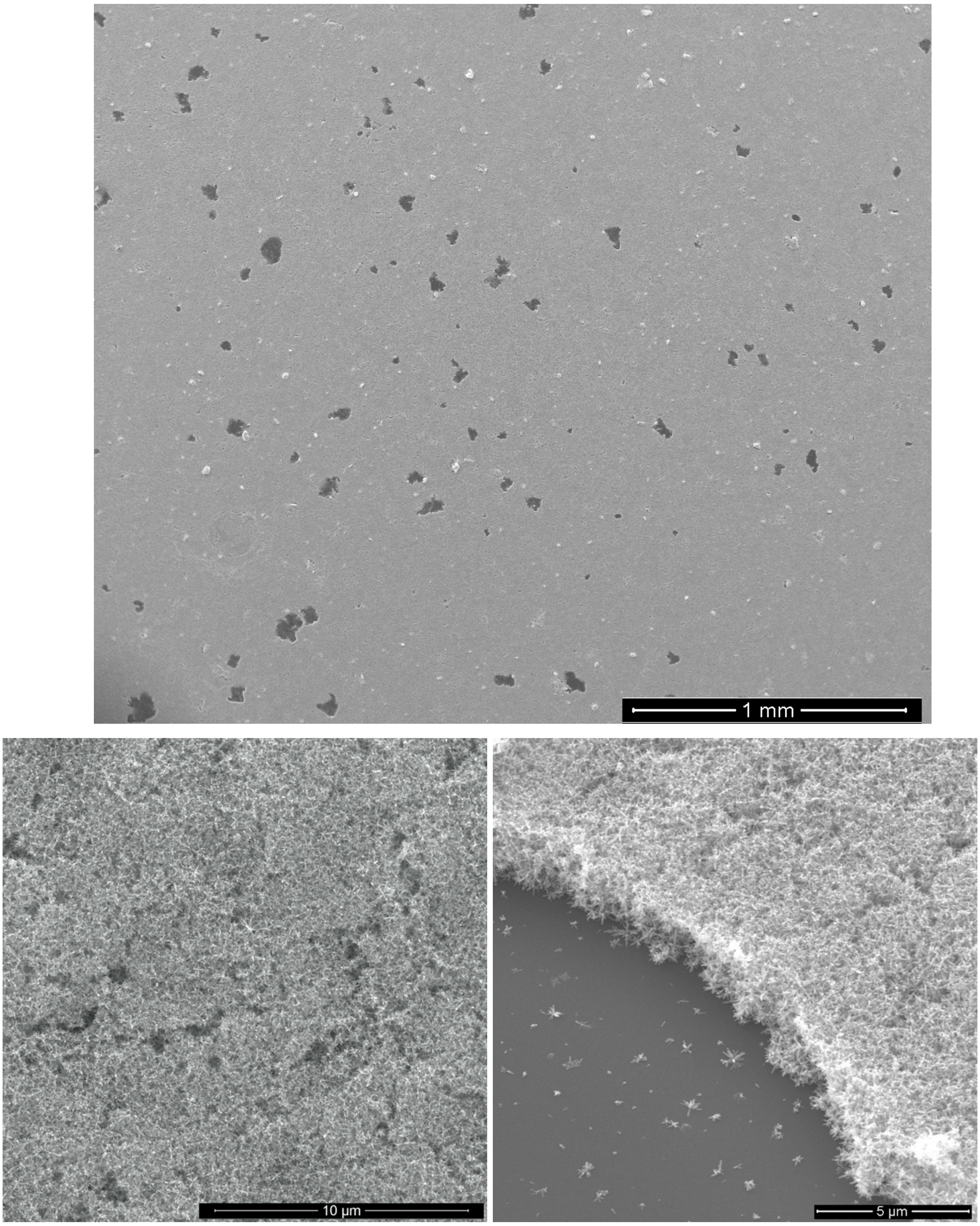

Figure S4. Scanning electron microscopy micrographs of AgNSs-coated glass surfaces.
